# The mechanisms of malic enzyme 2 in the tumorigenesis of human gliomas

**DOI:** 10.18632/oncotarget.9190

**Published:** 2016-05-05

**Authors:** Chiao-Pei Cheng, Li-Chun Huang, Yung-Lung Chang, Ching-Hsuan Hsieh, Shih-Ming Huang, Dueng-Yuan Hueng

**Affiliations:** ^1^ Graduate Institute of Medical Sciences, National Defense Medical Center, Taipei, Taiwan, ROC; ^2^ Department of Anesthesiology, Tri-Service General Hospital, National Defense Medical Center, Taipei, Taiwan, ROC; ^3^ Department of Biochemistry, National Defense Medical Center, Taipei, Taiwan, ROC; ^4^ Department of Neurological Surgery, Tri-Service General Hospital, National Defense Medical Center, Taipei, Taiwan, ROC

**Keywords:** glioblastoma multiforme, malic enzyme 2, reactive oxygen species, p53, tumorigenesis

## Abstract

The high level of resistance of glioblastoma multiforme (GBM) to currently used chemotherapies and other conventional therapies, its invasive characteristics and the presence of stem-like cells are the major factors that make the treatment of GBM difficult. Recent studies have demonstrated that the homeostasis of energy metabolism, glycolysis and mitochondrial oxidation of glucose are important for GBM cell growth and chemo-resistance. However, it is not clear which specific gene(s) are involved in the homeostasis of energy metabolism and invasiveness of GBM cells. We performed a preliminary analysis of data obtained from Gene Expression Omnibus profiles and determined that malic enzyme 2 (ME2) expression was positively associated with WHO grade in human primary gliomas. Hence, we evaluated the detailed working mechanisms of ME2 in human GBM cell processes, including proliferation, cell cycle, invasion, migration, ROS, and ATP production. Our data demonstrated that ME2 was involved in GBM growth, invasion and migration. ME2 has two cofactors, NAD^+^ or NADP^+^, which are used to produce NADH and NADPH for ATP production and ROS clearance, respectively. If the catalytic activity of ME2 is determined to be critical for its roles in GBM growth, invasion and migration, small molecule inhibitors of ME2 may be valuable drugs for GBM therapy. We hope that our current data provides a candidate treatment strategy for GBM.

## INTRODUCTION

The World Health Organization (WHO) classification proposes pathological grading of human gliomas from I to IV, including pilocytic astrocytomas (grade I), diffuse astrocytomas (grade II), anaplastic astrocytomas (grade III) and glioblastoma multiforme (GBM; grade IV) [1]. High-grade (grade III and IV) gliomas have unfavorable prognoses with high lethality due to recurrence after multimodal treatment, including surgical resection and radiation combined with chemotherapy. GBM is the most common and malignant primary brain tumor and has a median survival period of only 12-15 months; survival has not improved since the implementation of the current multimodal treatments [2]. The difficulty in treating GBM can be attributed to its high level of resistance to current chemotherapies and other conventional therapies and to its invasive characteristics and the presence of stem-like cells [3–5]. Recent studies have shown that homeostasis of energy metabolism, glycolysis and mitochondrial oxidation of glucose are important for GBM cell growth and chemo-resistance [6–10]. Therefore, there is an urgent need to identify specific gene(s) involved in the homeostasis of energy metabolism and invasiveness of GBM cells.

Altered cellular metabolism is a hallmark of cancer [11]. A growing number of studies have shown connections between oncogenic pathway members and molecules involved in cellular metabolism, such as glucose transporters, hexokinase, pyruvate kinase M2 and lactate dehydrogenase [12–16]. Recent studies showed that malic enzyme isoform 2 (ME2) might serve as a target for the suppression of tumor growth and invasiveness in several tumor cells, including lung cancer and melanoma cells [17–19]. MEs are oxidative decarboxylases that catalyze the oxidative decarboxylation of L-malate to pyruvate while simultaneously reducing NAD(P)^+^ to NAD(P)H [20, 21]. There are three different isoforms of MEs in mammalian tissues: a cytosolic NADP^+^ isoform (ME1), a mitochondrial NAD(P)^+^ isoform (ME2), and a mitochondrial NADP^+^ isoform (ME3). In addition to ME2, ME1 and ME3 also play important roles in physiologic and pathologic functions, such as insulin release and epithelial-mesenchymal transition (EMT) [22–26]. ME2 has two cofactors, NAD^+^ or NADP^+^; therefore, this enzyme may be key for rapidly proliferating cancer cells to meet their metabolic demands [18, 19]. However, the potential function of ME2 has not been thoroughly investigated in human gliomas.

Our preliminary analysis of data from Gene Expression Omnibus (GEO) profiles revealed that ME2 expression is positively associated with WHO grade in human primary gliomas, suggesting that ME2 may be, at least, a predictive biomarker in human gliomas. In this study, our laboratory evaluated the detailed mechanisms of ME2 in human GBM cell processes, including proliferation, cell cycle, invasion, migration, ATP production etc. We hope that our data are sufficient to support the functional roles of ME2 and lead to the development of a novel treatment strategy for GBM.

## RESULTS

### Depletion of endogenous ME2 levels by small hairpin RNA (shRNA) impaired cell proliferation and attenuated tumorigenic potential of GBM cells

**Figure 1:**
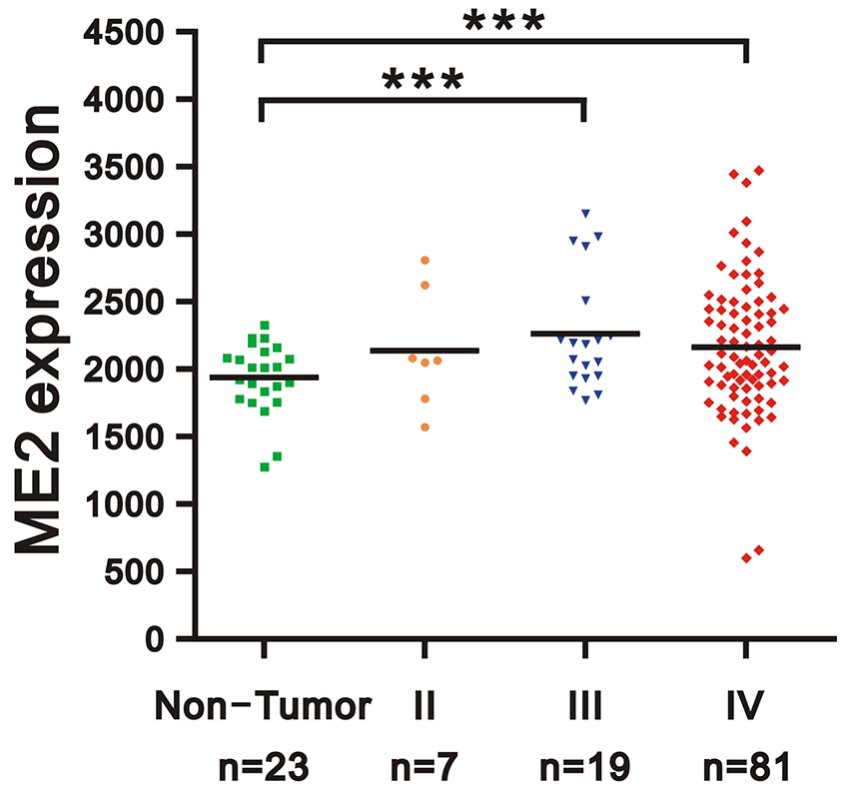
Expression level of ME2 in human gliomas of various stages Gene expression of ME2 in microarray datasets (GDS1962) of human gliomas: 26 cases of WHO grade II and III astrocytoma, including 7 cases of diffuse astrocytoma (WHO grade II), 19 cases of anaplastic astrocytoma (WHO grade III), and 81 cases of glioblastoma (WHO grade IV), compared with non-tumor controls. Two groups (benign and malignant) of data were analyzed using unpaired Student's t-test. (*** *P* < 0.001).

**Figure 2:**
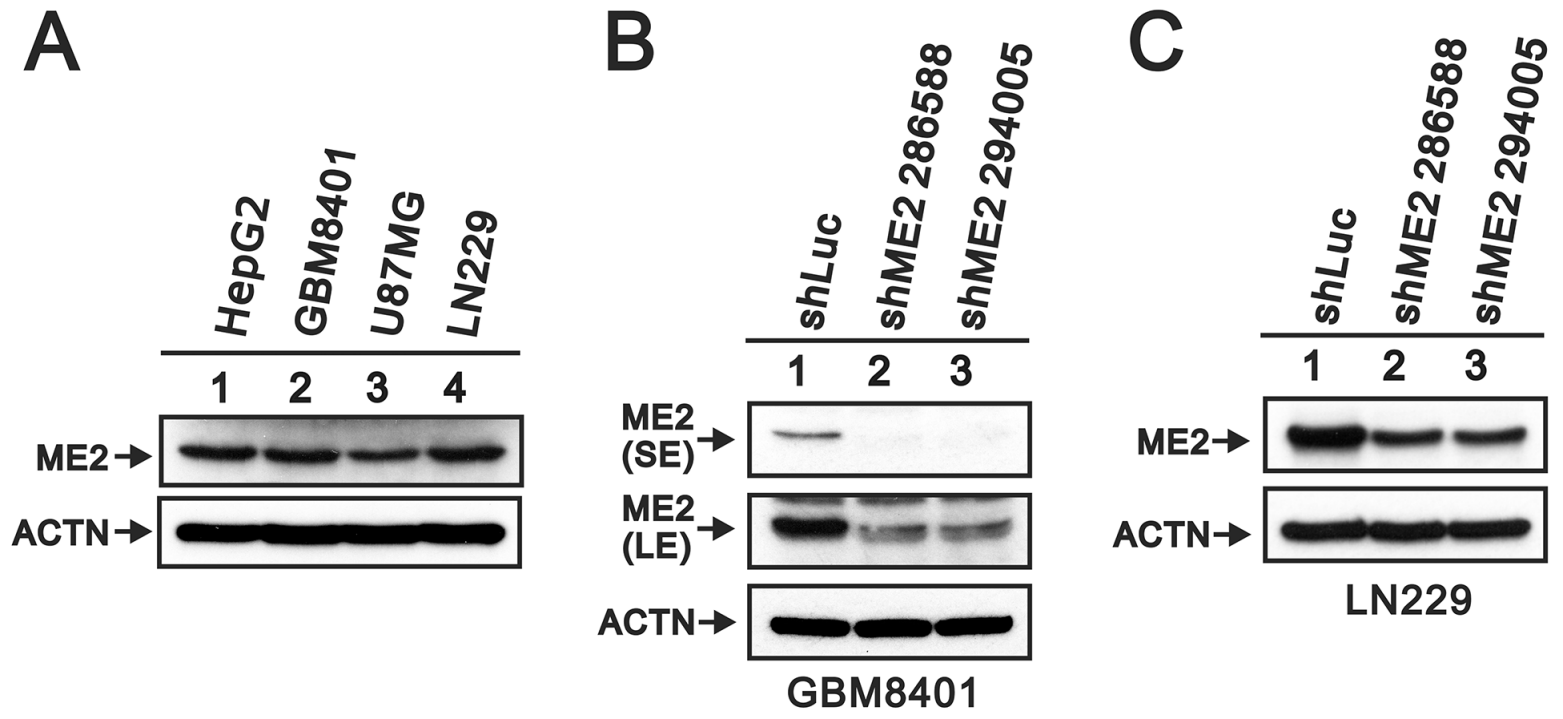
ME2 abundance in several human glioma cell lines **A.** Cells lysates (GBM8401, U87MG, LN229 glioma cells and positive control HepG2 hepatoma cells); **B.** GBM8401/shME2 (286588 and 294005); **C.** LN229/shME2 (286588 and 294005) and shLuc control cells were subjected to Western blotting with antibody against ME2 and ACTN antibody as the loading control. SE represents short exposure time, and LE represents long exposure time. Results are representative data from two independent experiments.

The GEO database showed that WHO pathological grading of human glioma and ME2 expression were associated. The ME2 mRNA expression levels in patient tissue samples were statistically greater in grade III and grade IV gliomas than in non-tumor controls (p<0.005) (Figure 1). Because ME2 mRNA expression was increased in the tumor samples, we first examined the ME2 protein level in three GBM cell lines, GBM8401, U87MG and LN229 [27], and the ME2 levels in all three GBM cell lines were comparative to those of the positive control, HepG2 cells (Figure 2A). Subsequently, we established two ME2 stable knockdown clones in GBM8401 and LN229 cells, GBM shME2 286588 and GBM shME2 294005. Compared with the GBM8401 shLuc control cells, the efficiency of the silencing of ME2 protein expression was over 90% in both stable knockdown clones (Figure 2B), however, the silencing of ME2 protein expression was around 70% in LN229 cells.

**Figure 3:**
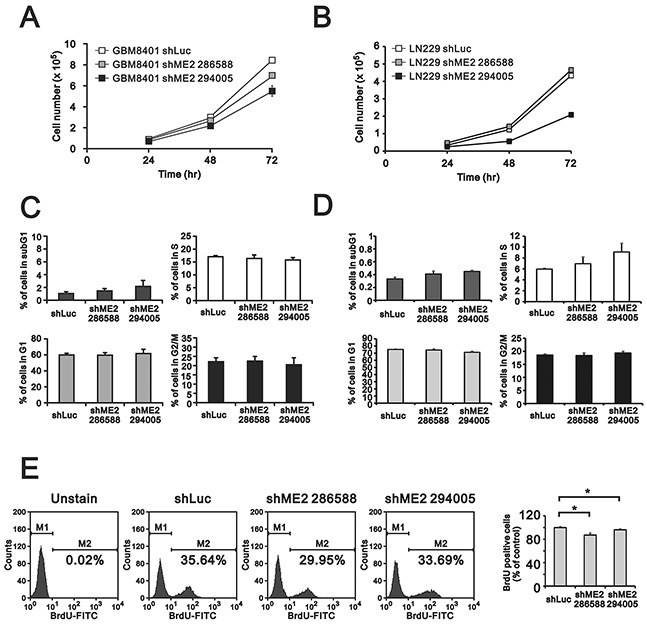
The effects of ME2 on cell proliferation and cell cycle profile in human glioma cell lines GBM8401 and LN229 shME2 cells were seeded (5×10^4^) in 12-well plates, and **A.** cell number were measured at the indicated time points; **B.** cell cycle analysis of GBM8401 and LN229 shME2 cells was determined by flow cytometry and **C.** BrdU incorporation by cells was performed using FITC BrdU Flow Kit. M1, BrdU-negative cells; M2, BrdU-positive cells. Cells untreated with BrdU was used as blank. The results of the statistical analysis are shown. Results are presented as the mean ± SD of triplicate samples from representative data of three independent experiments. (# p>0.05).

**Figure 4:**
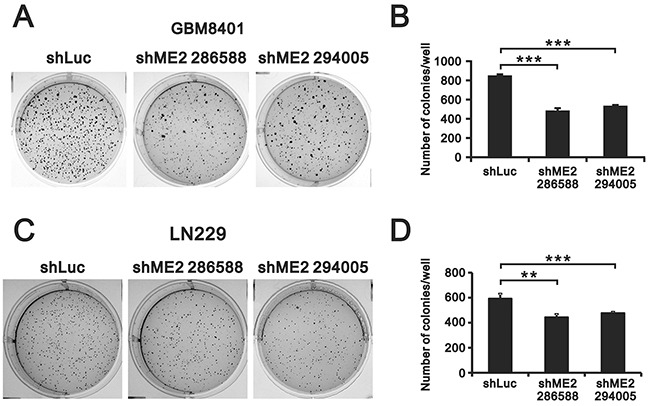
The effects of ME2 on soft-agar colony formation in human glioma cell lines Soft agar assays of GBM8401 and LN229 shME2 (286588 and 294005) and shLuc control cells are shown in **A.** and the statistical analysis is shown in **B.** Colonies > 0.5 mm were counted using ImageJ software. Results were presented as the mean ± SD of triplicate samples from representative data of three independent experiments. (*** *P* < 0.001).

Previous studies have found that ME2 affects cell proliferation [18, 19]. Hence, we examined the effects of ME2 silencing on cellular proliferation and colony formation in GBM8401 and LN229 cells. MTT assay revealed a lower cell number in GBM8401 shME2 cells than in shLuc control cells (Figure 3A), whereas one clone had the similar effect in LN229 shME2 cells (Figure 3B). The populations of cells in different cell cycle stages were not significantly different in either GBM8401 or LN229 shME2 clone compared to the shLuc control cells; however, the proportion of cells in subG1 phase was higher in the shME2 clones than in the shLuc control cells (Figure 3C and 3D). We also performed a BrdU assay, which showed that GBM8401 shME2 cells had relatively slower cellular proliferation rates than the shLuc control cells, suggesting that ME2 has an increasing role in cell proliferation (Figure 3E). A clonogenic assay in soft agar was used to study the effect of ME2 on cell survival (Figure 4A and 4C) the colony formation numbers of the two shME2 clones of GBM8401 and LN229 cells were significantly lower than that of the control cells (Figure 4B and 4D). In summary, our data suggest that ME2 may have a positive functional role in GBM cell growth.

### ME2 silencing increased GBM8401 cell invasion and migration

**Figure 5:**
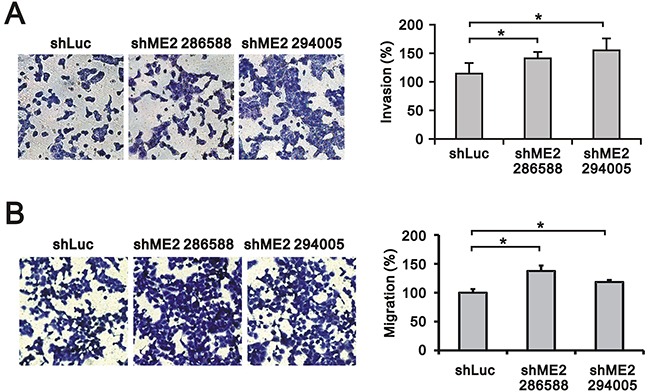
The effects of ME2 on invasion and migration in GBM8401 cells Invasion assay **A.** and migration assay **B.** of GBM8401 shME2 (286588 and 294005) and shLuc control cells are shown, and the statistical analysis was performed. Results were presented as the mean ± SD of triplicate samples from representative data of three independent experiments. (*P < 0.05).

Previous studies have shown that ME2 may participate in cancer cell invasion and migration processes [18, 19]. Our data showed that ME2 was important for anchorage-independent growth in GBM8401 cells. Hence, we further examined the functional role of ME2 in GBM8401 cell invasion using matrigel-based invasion and migration assays (Figure 5A and 5B). Our data suggest that ME2 is able to suppress cell invasion and migration in GBM8401 cell lines.

### ME2 silencing diminished ATP and reactive oxygen species (ROS) levels and increased lactate generation in GBM8401 cells

**Figure 6:**
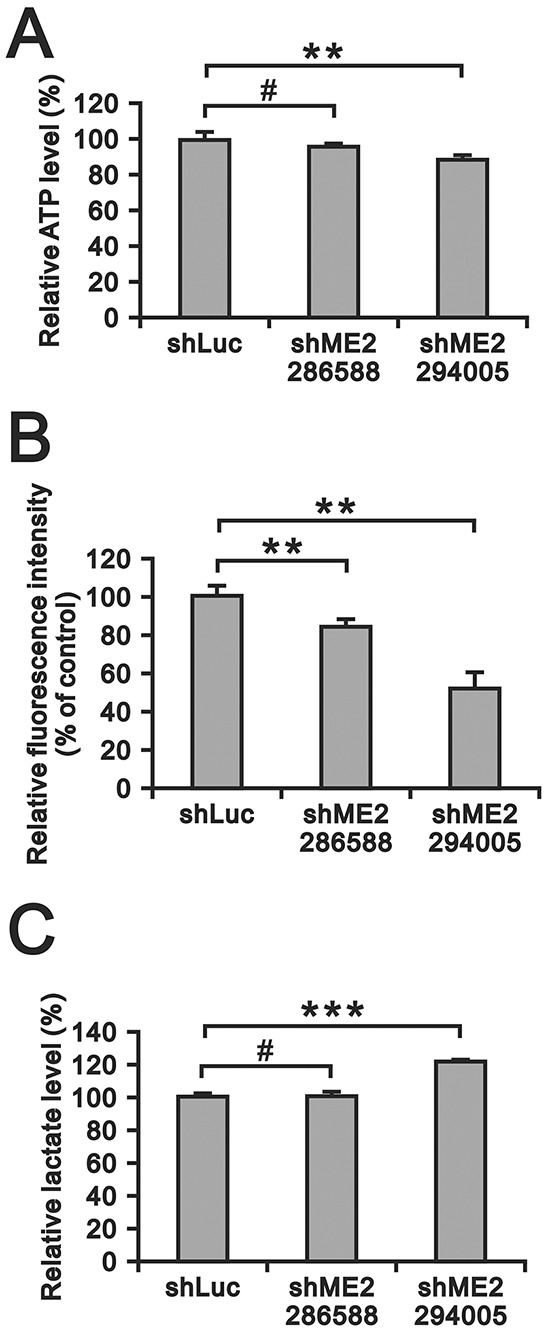
The effects of ME2 on ATP, ROS and lactate production in GBM8401 cells **A.** ATP levels in GBM8401 shME2 (286588 and 294005) and shLuc control cells were measured and normalized to the respective protein concentration. (**P < 0.01) **B.** ROS formation in GBM8401 shME2 (286588 and 294005) and shLuc control cells was detected by DCFH-DA staining. Cells were stained with DCFH-DA and measured by flow cytometry. Cells untreated with DCFH-DA were used as blanks. Results were presented as the mean ± SD of triplicate samples from representative data of three independent experiments. (**P < 0.01) **C.** Lactate production level in GBM8401 shME2 (286588 and 294005) and shLuc control cells were measured and normalized to the respective protein concentrations. Results are presented as the mean ± SD of triplicate samples from representative data of three independent experiments. (*** *P* < 0.001).

Otto Warburg hypothesized that the tumor cell metabolism involves an increased rate of aerobic glycolysis [28]. Tumor cells undergo aerobic glycolysis to accumulate biomass rather than ATP and evade apoptosis by releasing ROS from the mitochondria and producing lactate as a fuel source for tumor growth. Hence, we examined the effects of ME2 silencing on ATP, ROS and lactate production in GBM8401 cells. ATP production was significantly inhibited in GBM8401 shME2 (clone 294005) cells compared to GBM840 shLuc cells (Figure 6A). The basal ROS level was relatively high in GBM8401 shLuc cells, and ME2 silencing significantly diminished the ROS level (Figure 6B). We also observed that lactate production was increased in GBM8401 shME2 (clone 294005) cells (Figure 6C).

### The effects of ME2 knockdown on the ERK, PI3K/AKT and AMPK signaling pathways and EMT

**Figure 7:**
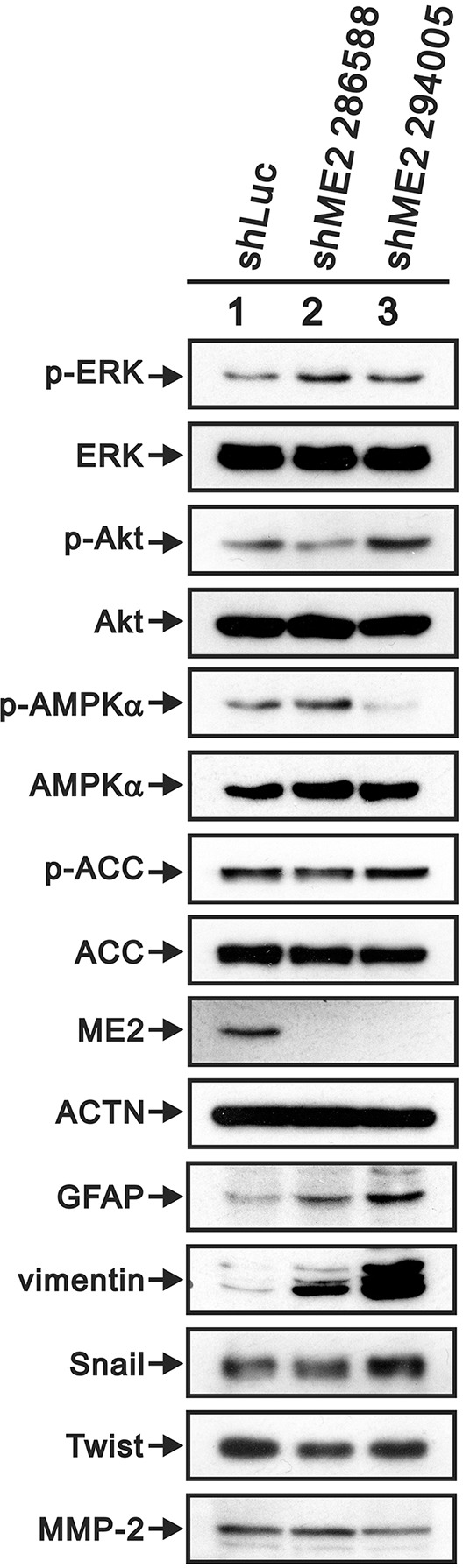
The effects of ME2 on the AMPK signaling and EMT pathways in GBM8401 cells Western blot analysis was utilized to determine the protein levels of AMPK signaling (p-ERK (Thr202/Tyr204), total ERK, p-Akt (Ser473), total Akt, p-AMPK (Thr172), total AMPK, p-ACC (Ser79) and total ACC) and EMT pathway markers (GFAP, vimentin, Snail, Twist and MMP-2) in GBM8401 shME2 (286588 and 294005) and shLuc control cells. ACTN was the loading control. Results are representative data of two independent experiments.

Metabolites interact with canonical signaling pathways [10, 29]. Epidemiological and experimental studies in human and animal cancer models have confirmed that the PI3K/AKT pathway is anomalously activated in cancer [30–33]. EGFR/PI3K/Akt signaling has been shown to be involved in the regulation of lipid metabolism in GBM [34]. Dr Chang's work demonstrated that AMPK activation is the mechanism for melanoma cell growth inhibition in response to ME2-depletion [19]. Therefore, we examined the status of ERK, AKT and AMPKα in GBM8401 cells. Our Western blot assay showed that depletion of ME2 resulted in increases in the levels of p-ERK and p-Akt and decreases in the levels of p-AMPKα and p-ACC (Figure 7).

Invasive growth is a phenotypic characteristic of EMT in GBM, and two EMT-promoting factors, Twist and Snail, are upregulated in high-grade gliomas compared to in low-grade gliomas [35]. Figure 7 presents the western blot data for GBM8401 shME2 cells: GFAP and vimentin protein levels were upregulated, and MMP-2 protein level was down-regulated. Furthermore, Twist, but not Snail, was down-regulated in GBM8401 shME2 cells.

### ME2 negatively regulates p53 functions in GBM cells

**Figure 8:**
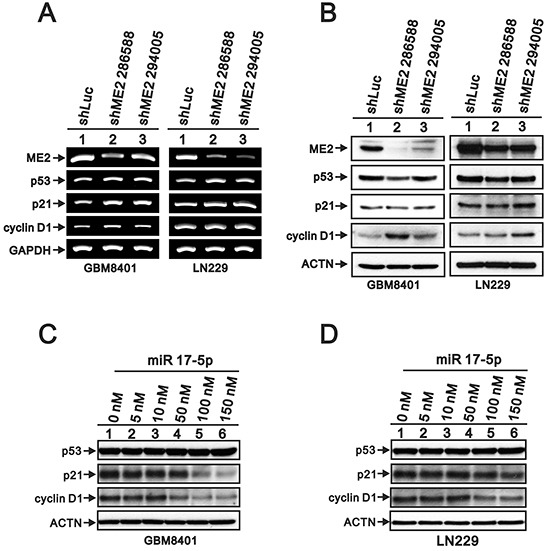
The effects of ME2 on the p53-dependent transcription functions in human glioma cell lines **A.** RT-PCR analysis of p53, p21 and cyclin D1 gene expressions in GBM8401 and LN229 shME2 (286588 and 294005) and shLuc control cells. GAPDH was the loading control. **B.** Western blot analysis of p53, p21 and cyclin D1 protein levels in GBM8401 and LN229 shME2 (286588 and 294005) cells and shLuc control cells. GBM8401 **C.** and LN229 **D.** cells were transfected with indicated amounts of miR 17-5p for 72 hrs and Western blot analysis was utilized to determine the protein levels of p53, p21 and cyclin D1. ACTN was the loading control. Results are representative data of two independent experiments.

A recent study showed that p53 and ME2 mutually negatively regulate each other, and the mechanism through which ME2 regulates p53 expression was also confirmed in melanoma [19, 36]. Here, we examined the effects of ME2 silencing on the expressions of p53 and its target gene, p21, using RT-PCR and Western blot (Figure 8). We observed increases in p53 and p21 mRNA in GBM8401 shME2 cells by RT-PCR (Figure 8A); however, the protein levels of p53 and p21 remained constant (Figure 8B). Cyclin D1 is a protein that is necessary for progression through the G1 phase of the cell cycle, and both its transcription and translation were induced by ME2 silencing (Figure 8A and 8B). We observed similar expression profile in LN229 cells (Figure 8A and 8B).

We further examined whether microRNAs (miRs) were involved into the regulation mechanism of ME2 in GBM cells. Our data revealed that eight miRs were increased and eight miRs were decreased in GBM8401 shME2 cells compared with shLuc cells by using the miR expression array analysis (Table 1). Based on the candidate target genes of these changed miRs, we tested whether miR 17-5p, one of the increased miRs under the ME2 knockdown condition, is responsible for the ME2-mediated modulation of p53, p21, and cyclin D1 expression in GBM8401 cells. MiR 17-5p did decrease the expression of its target genes, p21 and cyclin D1, not p53, in a dose-dependent manner, which supports that miR 17-5p functions well in GBM8401 cells (Figure 8). The inconsistency observed between ectopically expressing miR 17-5p and ME2 silencing on p21 and cyclin D1 (Table 1 and Figure 8), suggesting that miR 17-5p might not play the primary role in the regulation of p21 and cyclin D1 protein levels in response to ME2 ablation in GBM cells.

**Table 1:** miRs were modulated in ME2 knockdown GBM8401 cells

up-regulated miRs	down-regulated miRs
hsa-miR-100-5p	hsa-miR-513a-5p
hsa-miR-17-5p	hsa-miR-432-5p
hsa-miR-23a-3p	hsa-miR-30c-1-3p
hsa-miR-23b-3p	hsa-miR-2116-5p
hsa-miR-21-5p	hsa-miR-1587
hsa-miR-4638-5p	hsa-miR-4530
hsa-miR-7106-3p	hsa-miR-6870-5p
hsa-miR-6886-3p	hsa-miR-6779-5p

## DISCUSSION

There are three isoforms of MEs in mammals, and they have different cofactor specificities and subcellular localizations. In this study, we focused on the functions of ME2 in GBM cells, GBM8401 and LN229, based on the positive correlation between the expression level of ME2 and glioma grade. Hence, we down-regulated ME2 expression in GBM cells, and our findings suggest that ME2 might be involved in GBM cell growth, proliferation, metabolism, and invasion. GBM is a highly heterogeneous tumor that has been thoroughly profiled by the National Institutes of Health. We were interested in investigating whether ME2, in addition to epidermal growth factor receptor, contributes to GBM metabolic heterogeneity. ME2 has two cofactors, NAD^+^ and NADP^+^, which are used to produce NADH and NADPH for ATP production and ROS clearance, respectively. If the catalytic activity of ME2 is found to be critical for these aforementioned biological roles, small molecule inhibitors of ME2 may be valuable drugs for GBM therapy. Recent studies identified several small molecule inhibitors of ME2 enzymatic activity and found that they may induce cellular senescence [37, 38].

ROS can serve as both signaling molecules and cell death mediators to promote proliferation or induce cell death in response to chemotherapy in cancer [39, 40]. ROS are known to mainly be produced by oxidative phosphorylation during ATP synthesis, and the findings of the current study are consistent with this fact. However, previous study demonstrated that ME2 knockdown reduced ATP production and increased ROS level in melanoma cells [19]. This inconsistency may be due to differences in ROS clearance (reduced NADPH) and ROS generation (reduced ATP synthesis) when ME2 is depleted in GBM and melanoma cells, respectively. Hence, ME2-induced mitochondrial ROS may amplify the tumorigenic phenotype and accelerate the accumulation of additional mutations that lead to metastatic behavior through the induction of genomic instability in GBM cells.

Many studies have shown that the EMT is associated with tumor cell invasion leading to metastatic dissemination by promoting mesenchymal cell phenotypic characteristics, including enhanced migratory properties, invasiveness, and resistance to apoptosis [35, 41, 42]. In general, EMT is very complex and strictly controlled both temporally and spatially by the binding of several transcriptional repressors, including Twist, Snail and Slug, to the promoters of E-cadherin and a variety of EMT-related genes. Twist might play a key role in the survival and invasion of a subset of GBM tissues [35]. Our data showed that silencing ME2 expression consistently down-regulated Twist expression, suggesting that ME2 has a functional role in the EMT correlated with Twist expression. ME2 and p53 mutually negatively regulate each other, and down-regulation of Twist by ME2 silencing may be p53-dependent [36, 43]. It would be interesting to further investigate the functional roles of p53 and ME2 and their regulatory mechanisms in GBM.

The frustration in regards to the poor efficacy of the currently available GBM treatments and emerging evidence of the involvement of the homeostasis of energy metabolism, glycolysis and mitochondrial oxidation in GBM tumor progression encourage scientists to identify metabolic proteins that are potentially correlated with clinical survival. A number of glucose metabolic enzymes, such as, PKM2 and HK2, are attractive molecular targets [6, 7]. This work verified the functions of ME2 in GBM cells and indicated that ME2 may be a novel molecular target for drug development, and studies of currently identified specific ME2 inhibitors can be initiated [37, 38].

In summary, our findings demonstrate the mechanisms of ME2 in GBM cell proliferation, growth, invasion, migration and energy metabolism, specifically regarding its roles in ROS, ATP and lactate generation. ME2 is a promising target, and currently identified small molecular ME2 inhibitors can be examined to develop a novel GBM treatment strategy.

## MATERIALS AND METHODS

### Cell culture and RNA interference

Human GBM cells, GBM8401 and LN229 cells, were grown in DMEM medium supplemented with 10% fetal bovine serum, 100 units/ml penicillin and 100 mg/ml streptomycin. ME2-shRNA-containing lentiviral vectors and shLuc control vector were purchased from the National RNAi Core Facility (Academia Sinica, Taiwan, ROC). The procedural details are as previously described [44]. All cell lines were incubated at 37°C in 5% CO_2_.

### Immunoblot analysis

Cell lysates were harvested in lysis buffer (100 mM Tris–HCl pH 8.0, 150 mM NaCl, 0.1% SDS, and 1% Triton 100) at 4°C and were separated by SDS-PAGE electrophoresis. The proteins were then transferred to a polyvinylidine difluoride membrane (Millipore, USA) and were detected using antibodies against alpha-actinin (ACTN), p53, p21, cyclin D1, Twist, MMP-2 (Santa Cruz Biotechnology, USA), ERK, p-ERK, Akt, p-Akt, AMPKα, p-AMPKα, ACC, p-ACC, Snail (Cell signaling, USA), GFAP (BD Pharmingen, USA), vimentin (GeneTex Inc.), and ME2 (Sigma, USA).

### RT-PCR analysis

RNA was extracted using the total RNA reagent (Bioman, Taiwan, ROC). Then, 1.0 mg total RNA was subjected to reverse transcription using MMLV Reverse Transcriptase (Epicentre Biotechnologies, USA) according to the manufacturer's instructions. The forward and reverse PCR primers used are listed in Table 2 (p53, p21, cyclin D1, ME2, and GAPDH). The PCR products were subjected to 1.5% agarose gel electrophoresis and were visualized with UV light after ethidium bromide staining.

**Table 2:** Primers were used for PCR in this study

Gene Name	Primer Sequence (5′→3′)	Product Size
p53	Forward: GATGAAGCTCCCAGAATGCCAGAG	867 bp
	Reverse: GAGTTCCAAGGCCTCATTCAGCTC	
p21	Forward: CTGAGCCGCGACTGTGATGCG	345 bp
	Reverse: GGTCTGCCGCCGTTTTCGACC	
cyclin D1	Forward: ATGGAACACCAGCTCCTGTGCTGC	885 bp
	Reverse: TCAGATGTCCACGTCCCGCACGTCGG	
ME2	Forward: AGAGCTAGCCCAAGGGAGAC	410 bp
	Reverse: TCAACACGTCTACCCCAACA	
GAPDH	Forward: CTTCATTGACCTCAACTAC	486 bp
	Reverse: GCCATCCACAGTCTTCTG	

### Flow cytometry analysis of cell cycle and proliferation

The distributions of cells in different cell cycle stages were determined by measuring DNA content using fluorescence activated cell sorting (FACS). The cells were fixed in 70% ice-cold ethanol and kept at −20°C overnight. Before analysis, the harvested cells were washed with ice-cold PBS twice and stained with propidium iodide (PI) solution (5 mg/ml PI in PBS, 0.5% Triton x-100 and 0.5 mg/ml RNase A) for 30 min at 37°C in the dark.

For the proliferation analysis, the cells were treated and then processed with the FITC-BrdU Flow Kits according to the manufacturer's instructions (BD Biosciences). All samples were analyzed by the FACSCalibur flow cytometer (BD Biosciences). Data were analyzed by the Cell Quest Pro software (BD Biosciences).

### ATP and ROS measurement

Cellular ATP level was measured in GBM8401 cells (1×10^6^) using an ATP Colorimetric Assay Kit (BioVision, Milpitas, CA) according to the manufacturer's protocol. Intracellular ROS generation was measured by staining cells with 2′,7′- dichlorofluorescein-diacetate (DCFH-DA). Briefly, cells were incubated with 10 mM DCFH-DA for 30 min at 37°C. After the cells were harvested, they were washed twice with PBS and analyzed for 2′,7′-dichlorofluorescein (DCF) fluorescence using a FACSCalibur flow cytometer (BD Biosciences).

### Cell invasion and migration assays

GBM8401 cells (1×10^5^) were plated on the top wells of invasion chambers (BD Biosciences, San Jose, CA) that were coated with a thin layer of Matrigel matrix (specific for invasion assay). Then, cell invasion was induced by adding medium containing 5% serum to the bottom wells as a chemoattractant. The cells that transferred to the lower well of the chamber were stained using crystal violet.

### Soft agar assay

GBM8401 and LN229 cells (2.5×10^3^) suspensions were incubated in an upper layer of 0.35% agarose (Lonza Rockland, Inc.) in DMEM with 10% fetal bovine serum. The suspension was overlaid on 0.5% basal agar with 10% fetal bovine serum in a six-well plate and placed at room temperature until the agarose solidified. The plates were transferred to a 5% CO_2_ incubator and were incubated at 37°C for 2 weeks before being stained with crystal violet. Colonies larger than 0.5 mm in diameter were counted using ImageJ software (NIH, Bethesda, MD).

### MiRNA expression profiling

Total RNAs were extracted from shLuc and shME2 GBM8401 cells using the TRIsure (Bioline Reagents, London, UK) reagent and were subjected to Human miRNA OneArray (Phalanx Biotech Group, HsinChu, Taiwan, ROC). The RNA quantity and purity was assessed using a NanoDrop ND-1000. The pass criteria for the absorbance ratios are established at A260/A280≥1.8 and A260/A230≥1.5, indicating acceptable RNA purity. The RIN values were ascertained using the Agilent RNA 6000 Nano assay to determine the RNA integrity. The pass criteria for the RIN value were established at ≥6, indicating acceptable RNA integrity. gDNA contamination was evaluated by gel electrophoresis. The data were analyzed with the Rosetta Resolver® System (Rosetta Biosoftware, WA, USA).

### Statistical analysis

Statistical values are expressed as the means ± SD of at least three independent experiments. All comparisons between groups were performed using the unpaired two-tailed t-test. Statistical significance was set at *p*<0.05.

## Abbreviations

GBM: glioblastoma multiforme
GEO: Gene Expression Omnibus
ME: malic enzyme
miR: micro RNA
FACS: fluorescence activated cell sorting
PI: propidium iodide
DCFH-DA: 2′,7′-dichlorofluorescein-diacetate
DCF: 2′,7′-dichlorofluorescein.
